# Proximity Sensing Electronic Skin: Principles, Characteristics, and Applications

**DOI:** 10.1002/advs.202308560

**Published:** 2024-01-28

**Authors:** Bingwei Wu, Ting Jiang, Zhongxiang Yu, Qihui Zhou, Jian Jiao, Ming Liang Jin

**Affiliations:** ^1^ Heart Center, Qingdao Hiser Hospital Affiliated of Qingdao University Qingdao University Qingdao 266033 China; ^2^ Institute for Future, Shandong Key Laboratory of Industrial Control Technology, School of Automation Qingdao University Qingdao 266071 China; ^3^ School of Rehabilitation Sciences and Engineering University of Health and Rehabilitation Sciences Qingdao 266000 China; ^4^ Peng Cheng Laboratory Shenzhen 518055 China

**Keywords:** proximity sensing electronic skin, human‐robot collaboration, human‐machine interface, remote monitoring

## Abstract

The research on proximity sensing electronic skin has garnered significant attention. This electronic skin technology enables detection without physical contact and holds vast application prospects in areas such as human‐robot collaboration, human‐machine interfaces, and remote monitoring. Especially in the context of the spread of infectious diseases like COVID‐19, there is a pressing need for non‐contact detection to ensure safe and hygienic operations. This article comprehensively reviews the significant advancements in the field of proximity sensing electronic skin technology in recent years. It covers the principles, as well as single‐type proximity sensors with characteristics such as a large area, multifunctionality, strain, and self‐healing capabilities. Additionally, it delves into the research progress of dual‐type proximity sensors. Furthermore, the article places a special emphasis on the widespread applications of flexible proximity sensors in human‐robot collaboration, human‐machine interfaces, and remote monitoring, highlighting their importance and potential value across various domains. Finally, the paper provides insights into future advancements in flexible proximity sensor technology.

## Graphical abstract

This article focuses on the research progress of capacitive proximity sensors and triboelectric proximity sensors in terms of the characteristics. Then, the applications of the flexible proximity sensors are introduced from three aspects: human‐robot collaboration, human‐machine interface, and remote monitoring. And discussed the challenges of flexible proximity sensors in the future.

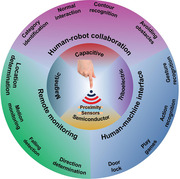

## 1 Introduction

The skin is the largest sensory organ in humans, serving multiple functions such as protection and sensation.^[^ ^1^, ^2^, ^3^ ^]^ It functions as the body's paramount natural defense, characterized by exceptional attributes such as impressive stretchability, self‐healing capabilities, excellent mechanical toughness, and sensitive tactile perception. Structurally, the skin can be roughly divided into three layers: the epidermis, dermis, and subcutaneous tissue.^[^ ^4^ ^]^ In addition to these three layers, there are also vellus hairs on the skin's surface. In daily life, each vellus hair serves as numerous detectors, converting signals from foreign objects that have not made physical contact with the skin into stimuli and relaying them to the nerve cells beneath the skin, thereby alerting the body. Electronic skin possesses human‐like pressure sensitivity,^[^ ^5^, ^6^, ^7^, ^8^, ^9^, ^10^ ^]^ pain perception,^[^ ^11^, ^12^, ^13^ ^]^ and temperature sensing,^[^ ^14^, ^15^ ^]^ and additionally, it encompasses functions that exceed those of human skin, including proximity detection^[^ ^16^, ^17^, ^18^ ^]^ and chemical sensing.^[^ ^19^, ^20^ ^]^

The advent of flexible electronics has ushered in limitless prospects in the realm of smart electronics, encompassing flexible touch screens^[^ ^21^ ^]^ and implantable devices.^[^ ^22^ ^]^ However, flexible sensors hold greater value in the realization of flexible wearable devices. Flexible sensing represents a crucial research focus in the field of flexible electronics. Based on the mode of contact, sensors can be divided into contact and non‐contact sensors. Currently, the literature mainly reports on contact sensors, but non‐contact sensors actually have advantages in specific situations.

A proximity sensor device is used to detect the proximity of objects, enabling non‐contact detection. The proximity of an object causes a change in the sensor's signal. Proximity sensing serves as a complement to both vision and touch.^[^ ^23^ ^]^ For touch sensors, proximity sensors provide perception before physical contact, while for visual sensors, they compensate for limitations related to close‐range blind spots.

The rapid development of information systems has made the human‐machine relationship increasingly important.^[^ ^24^ ^]^ Robots play substantial roles in our lives. In conjunction with the industrial revolution and the shifting paradigm of manufacturing, the evolving human‐machine relationship is delineated by the 5C journey: Coexistence, Cooperation, Collaboration, Compassion, and Coevolution.^[^ ^25^ ^]^ Technological progress empowers robots to engage with humans across various domains.^[^ ^26^, ^27^ ^]^ Safety is the paramount concern in the hierarchy of human needs within the industrial context. Ensuring human safety represents the foremost priority in a manufacturing environment. Traditional industrial robot systems necessitate robust fencing and peripheral safety devices,^[^ ^28^ ^]^ or rely on reactive protection.^[^ ^29^ ^]^ However, fencing diminishes flexibility while inflating costs and space requirements, and reactive protection poses the risk of harm to either the robotic arm or humans. Protective mechanisms should evolve toward proactive protection.^[^ ^30^ ^]^ Moreover, microbial pathogens pose a persistent threat to humans. In a span of just a few decades in the 21st century, the world has experienced three significant pandemics: SARS, MERS, and the recent COVID‐19.^[^ ^31^, ^32^ ^]^ COVID‐19 can be transmitted through direct contact with surfaces used by infected individuals.^[^ ^33^ ^]^ In everyday life, numerous diseases may also spread through contact, including hand, foot, and mouth disease, and dysentery, among others. Additionally, traditional rigid proximity sensors typically cannot achieve large‐scale and ubiquitous detection, and their compact and rigid appearance limits their application in these areas.^[^ ^34^ ^]^ Visual sensors have problems with large data processing and visual blind spots. Therefore, flexible proximity sensors hold substantial development potential, whether in the realm of human‐robot collaborative safety or in mitigating virus transmission through non‐contact methods in daily life.

**Figure 1:**
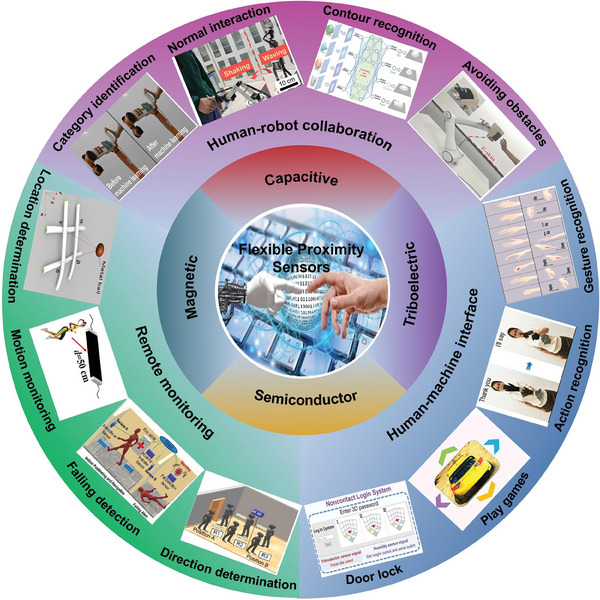
Classification and application of flexible proximity sensors. Middle: By [geralt] via Pixabay. Category identification: Reproduced under terms of the CC‐BY license.^[^ ^110^ ^]^ Copyright 2023, The Authors, published by Wiley‐VCH. Gesture recognition: Reproduced with permission.^[^ ^127^ ^]^ Copyright 2022, Wiley‐VCH. Action recognition: Reproduced with permission.^[^ ^137^ ^]^ Copyright 2021, American Chemical Society. Motion monitoring: Reproduced with permission.^[^ ^141^ ^]^ Copyright 2020, American Chemical Society. Door lock and Play games: Reproduced under terms of the CC‐BY license.^[^ ^138^ ^]^ Copyright 2022, The Authors, published by Wiley‐VCH. Avoiding obstacles: Reproduced under terms of the CC‐BY license.^[^ ^79^ ^]^ Copyright 2022, The Authors, published by Wiley‐VCH. Contour recognition and Normal interaction: Reproduced under terms of the CC‐BY license.^[^ ^108^ ^]^ Copyright 2022, The Authors, published by AAAS. Falling detection and Direction determination: Reproduced with permission.^[^ ^140^ ^]^ Copyright 2021, Elsevier. Location determination: Reproduced under terms of the CC‐BY license.^[^ ^37^ ^]^ Copyright 2020, American Chemical Society.

The structure of this paper is as follows: First, the principles of various types of proximity sensors are introduced, including capacitive, triboelectric, semiconductor, and magnetic sensors. The third section focuses on the characteristics such as large area, multifunctionality, strain, and self‐healing, providing a detailed overview of the research progress in single‐type flexible proximity sensors. Then, the advancements in dual‐type proximity sensors are discussed, further enriching the development direction of flexible proximity sensors. In the section highlighting the applications of flexible proximity sensors, particular attention is given to their practical use in areas such as human‐robot collaboration, human‐machine interfaces, and remote monitoring. These practical examples vividly demonstrate the substantial and indispensable role of flexible proximity sensors in diverse fields. Finally, in the conclusion section, a comprehensive summary and summary of the research results of this article is provided, emphasizing the potential and future prospects of flexible proximity sensors (**Figure** **1**).

## 2 Transduction Mechanisms

Categorized by their sensing principles and mechanisms, flexible proximity sensors encompass various types, including capacitive, triboelectric, semiconductor, and magnetic field, among others. **Table** **1** provides an overview of the advantages and disadvantages of various types of flexible proximity sensors. Of these, capacitive sensing and triboelectric sensing have garnered substantial attention in academic literature. In this section, we primarily focus on flexible proximity sensors based on capacitive and triboelectric sensing principle.

**Table 1:** Comparison of different types of flexible proximity sensors.

Type	Advantages	Disadvantages
Capacitive	Low cost, flexible structural design, and fast dynamic response	Parasitic capacitance and susceptibility to interference from the surrounding environment (such as electromagnetic interference)
Triboelectric	Simple structure, self‐power	Usually output transient signal, charge attenuation, susceptible to environmental interference
Semiconductor	Strong ability to detect insulators, high accuracy, and simple structure	Restricted object detection, usually detecting insulators
Magnetic	Not affected by non‐magnetic objects, allowing for accurate signal acquisition	Restricted object detection, detecting magnetic objects

### 2.1 Capacitive Proximity Sensor

**Figure 2:**
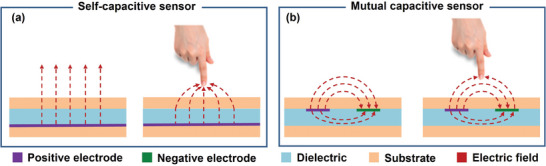
Two types of capacitive proximity sensor principles. a) Self‐capacitive sensor. b) Mutual capacitive sensor.

According to the capacitance detection principle, capacitive proximity sensors can be divided into two categories: self‐capacitance proximity sensor^[^ ^35^ ^]^ and mutual capacitance proximity sensor.^[^ ^36^ ^]^ As shown in **Figure** **2**a, self‐capacitance refers to the capacitance between the sensing electrode and the ground (commonly referred to as “GND” in the circuit). The application of an excitation signal to the electrode induces a changing electric field between the electrode and the ground, owing to the presence of self‐capacitance. As an object approaches, the parasitic capacitance to the ground undergoes alterations, enabling the detection of the object. On the other hand, as shown in Figure 2b, mutual capacitance entails two electrodes: the transmitting electrode and the receiving electrode. An electric field is established between the two electrodes. While the majority of the energy is concentrated between the capacitor plates, a small portion escapes into the surrounding space beyond the electrode plates. These escaping electric fields are commonly termed “fringe fields”.

In the course of approaching the sensor using mutual capacitance, two capacitors come into play: the mutual capacitance between the two electrodes and the self‐capacitance to ground, often referred to as fringe capacitance. When an object approaches, the hand serves as a third electrode capable of partially intercepting the fringe electric field and redirecting it to the ground within the sensor's sensing range. Throughout this process, charges flow out from both electrodes, resulting in alterations in fringe capacitance and mutual capacitance.^[^ ^37^ ^]^

Mutual capacitive proximity sensors typically employ a sandwich structure comprising electrodes and dielectric layers. The materials and structure of the device are pivotal in determining the strength and distribution of the fringe field, consequently influencing the overall performance of the sensor. Research has demonstrated that several factors, including electrode shape and parameters, significantly influence performance metrics such as signal strength, sensitivity, and signal‐to‐noise ratio for capacitive proximity sensors. Presently, extensive research has been conducted on electrode shapes for capacitive proximity sensors, including structures like the parallel plate structure,^[^ ^38^ ^]^ interdigital electrode,^[^ ^39^, ^40^, ^41^, ^42^, ^43^, ^44^, ^45^, ^46^ ^]^ innovative complementary Archimedean spiral electrode,^[^ ^47^ ^]^ circular‐circular configuration,^[^ ^48^ ^]^ and crossed electrodes,^[^ ^37^, ^49^ ^]^ among others. These structural variations alter the effective area between the electrodes, thereby impacting the sensor's sensitivity and other performance aspects. **Table** **2** provides an overview of the research results related to flexible proximity sensors using interdigital electrodes.

**Table 2:** Summary of the interdigital electrode in proximity sensors.

Electrode structure	Electrode material	Tested object	Detection distance(mm)	Refs.
Interdigital Electrode	LM	Human hand	50	[46]
Interdigital Electrode	ITO	Human hand	200	[39]
Annular Interdigital Electrode	Cu	Human hand	NA	[45]
Interdigital Electrode	Au	Acrylic acid	1.4	[44]
Interdigital Electrode	EGaIn	Human hand	150	[42]
Interdigital Electrode	CNTs	Force gauge probe	2	[41]
Spider Web Electrode	EGaIn	Human hand	5	[43]
Interdigital Electrode	Cu	Human hand	100	[40]

### 2.2 Triboelectric Proximity Sensor

**Figure 3:**
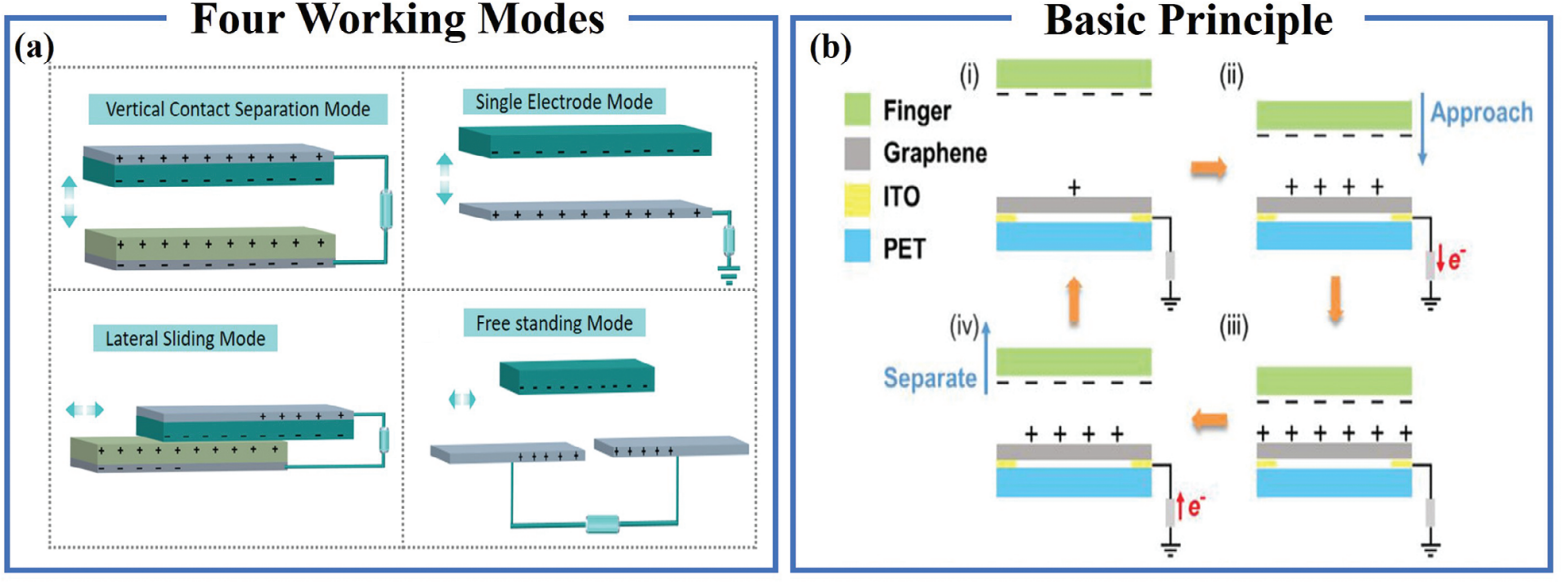
Triboelectric nanogenerators and triboelectric proximity sensors. a) TENG four operating modes. Reproduced with permission.^[^ ^52^ ^]^ Copyright 2022, Wiley‐VCH. b) Principle of triboelectric proximity sensor. Reproduced with permission.^[^ ^55^ ^]^ Copyright 2019, Wiley‐VCH.

The phenomenon of triboelectricity has been a topic of interest in the field of tribology for centuries. For over 2000 years, the universality and intriguing aspects of frictional electricity have captured the attention of researchers.^[^ ^50^ ^]^ The triboelectric nanogenerator (TENG), first pioneered by Zhonglin Wang and his team in 2012, is designed to convert minute mechanical energy into electrical energy through the exploitation of the triboelectric charging effect and electrostatic induction effect.^[^ ^51^ ^]^ TENG operates in four distinct modes: vertical contact mode, single‐electrode mode, lateral sliding mode, and independent friction layer mode [**Figure** **3**a].^[^ ^52^, ^53^ ^]^ While most TENG devices are currently utilized as self‐powered sensors for various mechanical movements, they can also function as proximity sensors.^[^ ^54^ ^]^ Tang et al. first proposed the concept of non‐contact induction based on the participation of the human body in frictional electric self‐power supply.^[^ ^55^ ^]^

The operational principle of triboelectric proximity sensors, founded on triboelectricity and electrostatic induction, is depicted in Figure 3b. In the initial state, when two objects are separated, the lower surface of one object induces a charge, such as positive charge, while the upper object induces a negative charge. During the approach phase, as the upper object nears, the lower object undergoes electrostatic induction, accumulating extra positive charges to neutralize the charges on the upper object. This process results in the flow of electrons through the electrode toward the ground, generating a current signal. In the phase of electrostatic equilibrium, as the objects draw nearer, the charges produced by the upper object are entirely neutralized by those generated by the lower object, resulting in electrostatic equilibrium. In the departure phase, as the upper object retreats from the sensor, the positive charges on the electrode diminish, and free electrons are attracted to the lower electrode to counterbalance the positive charges, leading to a current in the opposite direction to that during the approach phase. In daily life, owing to the triboelectric effect between footwear and the ground, the human body retains the electrical charge. Moreover, negative charges may accumulate at the fingertips due to the tip effect. Consequently, via electrostatic induction, flexible sensors based on the triboelectric effect can detect the proximity of objects, including a human hand.^[^ ^55^ ^]^

However, proximity sensors based on triboelectricity also face challenges similar to those encountered by capacitive proximity sensors—a decrease in output performance as the distance from the object increases. To enhance output performance, a series of methods are typically employed, including material optimization, structural design, and the use of ultra‐thin dielectric layers, among others, to increase surface charge density. These measures contribute to the improvement of the sensor's performance.^[^ ^56^ ^]^ For example, Chen and colleagues utilized processes such as oxygen plasma etching, ECR sputtering, and through‐filter ion etching to establish a secondary micro‐nano composite structure on the sensing layer's surface. This approach substantially increased the specific surface area and enhanced the sensor's output signal by ≈200%.^[^ ^57^ ^]^ Similarly, Zhang et al. harnessed the high specific surface area and structural stability of spherical multiple physical networks to produce composite films of polyvinylidene fluoride (PVDF) @Mxene (Ti_3_C_2_T_x_) with multiple physical net‐work structures for use as triboelectric materials. Ti_3_C_2_T_x_ material and spherical multiple physics network structure play a great role in improving TENG output. The H and O atoms of Ti_3_C_2_T_x_ and the H atoms in the PVDF chain form dipoles. The capacitor network formed by the dipoles and porous microstructure improves the output capability of the TENG. The size of the porous microspheres in the spherical multiple physical network structure can change the effective contact area. When the diameter of the microsphere is 6.536 µm, the output charge density can reach 128 µC m^−2^, and the instantaneous output power can reach 200 µW cm^−2^.^[^ ^58^ ^]^

### 2.3 Semiconductor and Magnetic Proximity Sensors

**Figure 4:**
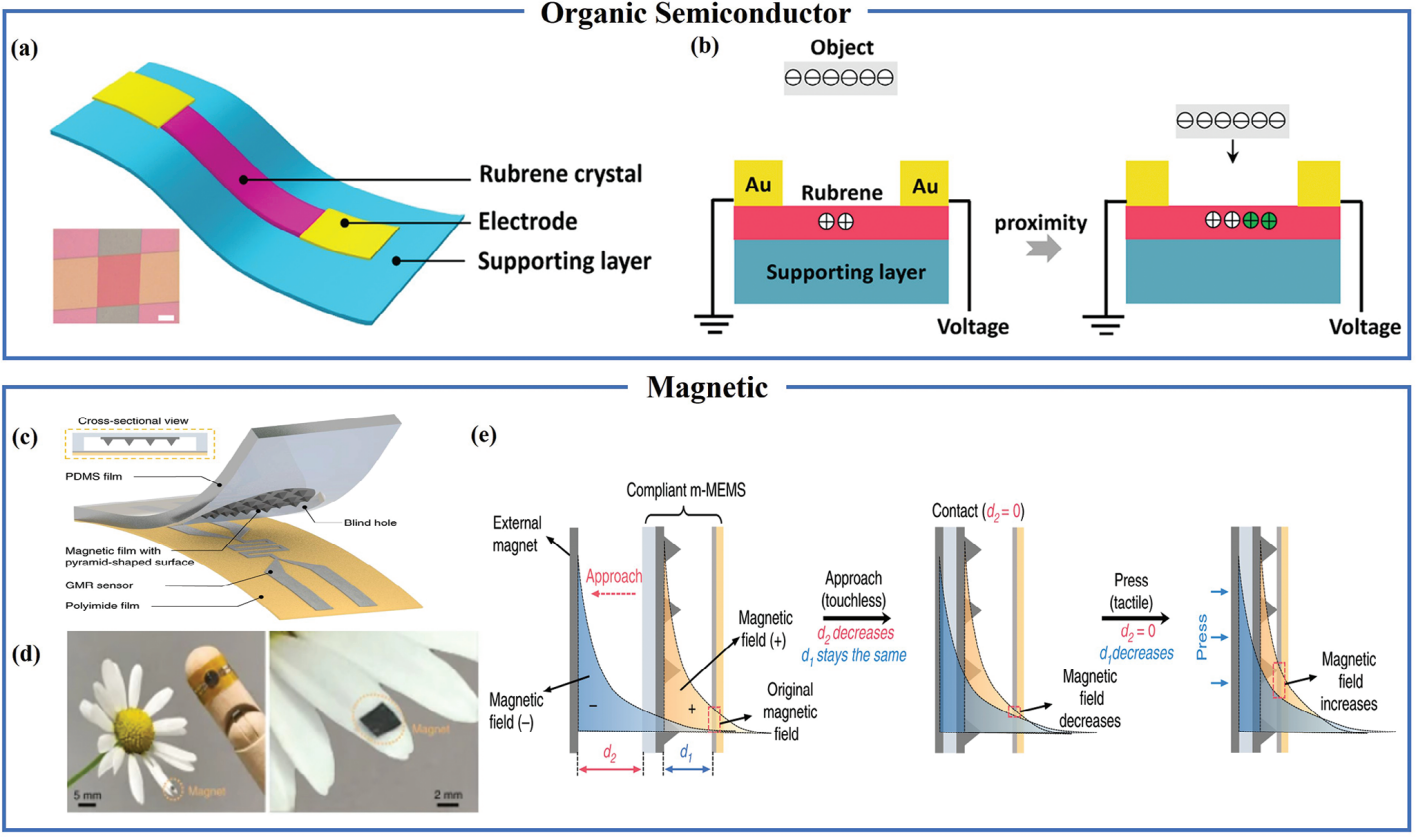
Semiconductor and magnetic proximity sensors. a) Structural diagram of flexible proximity sensor based on ultra‐thin rubrene micro nano single crystal. b) Schematic diagram of flexible proximity sensor based on ultra‐thin rubrene micro nano single crystal. Reproduced with permission.^[^ ^59^ ^]^ Copyright 2018, American Chemical Society. c) m‐MEMS platform. d) Daisy petals decorated with permanent magnets. e) Schematic diagram of the evolution of magnetic field intensity at the sensor position: the superposition of the builtin magnetic field provided by a compatible permanent magnet (orange shaded area) and an external magnetic field source (blue shaded area). Reproduced under terms of the CC‐BY license.^[^ ^63^ ^]^ Copyright 2019, The Authors, published by Springer Nature.

Flexible semiconductor proximity sensors differ from traditional capacitive sensors. These sensors have electrodes located at the two ends of semiconductor material, creating a two‐terminal planar device structure. Wang et al. pioneered the use of a flexible rubrene crystal as a sensing element, achieving a sensor with proximity sensing capability, as shown in **Figure** **4**a, where gold electrodes are located at both ends of the micro‐sized organic single crystal. When a charged object approaches the sensor, the electric field produced by the charges, due to electrostatic induction, serves as the gate voltage, capable of modulating the charge carriers within the organic semiconductor. This modulation leads to a current alteration in the organic device, as depicted in Figure 4b.^[^ ^59^ ^]^ Most sensors based on this principle employ semiconductor materials as the sensing layer, such as DPP‐DTT,^[^ ^60^ ^]^ rGO film,^[^ ^61^ ^]^ and DNTT.^[^ ^62^ ^]^ These sensors are primarily used for detecting non‐metallic objects with static electricity.

Recent research suggests that magnetic‐based proximity sensors can address some of the challenges faced by capacitive proximity sensors, such as issues related to interference from unrelated objects and the identification of signal sources. Magnetic‐based flexible proximity sensors frequently employ principles such as the Hall effect, magneto‐piezoresistive effect, and giant magnetoresistance effect for object proximity detection. The hall effect refers to the phenomenon in a conductor where, when an electric current flows, an electric field is generated perpendicular to both the current direction and the magnetic field direction, resulting in a lateral voltage. Magnetoresistance effect is the phenomenon in which the resistance of certain metals or semiconductors changes with the variation of an applied magnetic field. Giant magnetoresistance effect refers to the phenomenon where the resistivity of magnetic materials undergoes a significant change when subjected to an external magnetic field as compared to when there is no external magnetic field. Ge et al. incorporated flexible permanent magnet NdFeB particles into PDMS rubber, forming a magnetic micro‐electromechanical system (m‐MEMS) as depicted in Figure 4c. In Figure 4d, magnetic material opposite to the flexible permanent magnet is applied to the object to induce specific object detection. Figure 4e illustrates the interaction process, wherein the external magnetic field compensates for the built‐in magnetic field when in proximity, leading to an increase in GMR (giant magnetoresistance) resistance. Upon contact, the magnetic field at the GMR sensor's location increases, causing a reduction in GMR resistance.^[^ ^63^ ^]^

## 3 Single Type Proximity Sensor

A single type of proximity sensor uses a single proximity sensing principle to detect objects. For example, capacitive proximity sensors and triboelectric proximity sensors. In this section, we concentrate on the research advancements in single type proximity sensors from four dimensions: large area, multifunctionality, strain, and self‐healing.

### 3.1 Large Area

**Figure 5:**
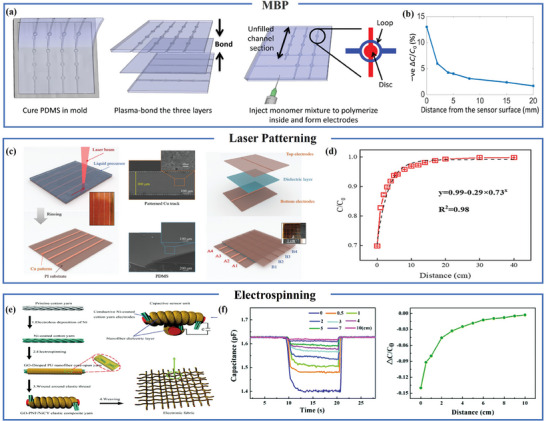
Manufacturing process based on microfluidics, laser patterning, and electrospinning for large‐area flexible proximity sensors. a) Sensor manufacturing process. b) Relative variation of capacitance near fingers. Reproduced under terms of the CC‐BY license.^[^ ^66^ ^]^ Copyright 2017, published by AAAS. c) Manufacturing process of microscale components of sensors. d) Variation of relative capacitance of proximity sensors with finger distance. Reproduced with permission.^[^ ^67^ ^]^ Copyright 2020, Wiley‐VCH. e) Schematic diagram of electronic fabric manufacturing. f) Proximity response of elastic composite yarn. Reproduced with permission.^[^ ^68^ ^]^ Copyright 2018, Royal Society of Chemistry.

One of the key characteristics of the skin is its substantial surface area. The skin serves as the boundary between the human body and the external environment, constituting the largest organ in the human body and making up roughly 15% of the total body mass.^[^ ^64^, ^65^ ^]^ When considering the utilization of flexible proximity sensors in applications such as human‐robot collaboration, the imperative of large‐scale production becomes evident. Currently, diverse methods are available for the fabrication of electronic skin, encompassing techniques such as photolithography and solution‐based processes. In 2017, Sarwar and colleagues implemented a mold‐peel‐bond technique, similar to the fabrication process for microfluidic devices, for the development of integrated sensors. The manufacturing process, depicted in **Figure** **5**a, involves pouring PDMS into a mold, followed by peeling and bonding, and ultimately injecting ion‐conductive hydrogel (consisting of acrylamide and NaCl) into the circular disk and ring structure of PDMS. This sensor demonstrates a detection distance of 12 cm [Figure 5b].^[^ ^66^ ^]^ Li and his research team employed laser technology to create copper electrode patterns on a polyimide film and a polydimethylsiloxane (PDMS) medium, which were subsequently used in the assembly of both single pixel touch sensors and multi‐channel sensors. The structure and manufacturing process for these sensors are elucidated in Figure 5c, encompassing precursor preparation, laser patterning, and assembly, all of which can be completed within an hour. The definition of patterns and annealing take place during the repetitive laser scanning process. While the single‐pixel proximity sensor achieves a detection distance of up to 20 cm [Figure 5d], the multi‐channel sensor exhibits a relatively reduced detection distance and requires further optimization.^[^ ^67^ ^]^ You et al. applied a coating of graphene oxide (GO)‐doped polyurethane (PU) nanofibers to the surface of Ni‐coated cotton yarn using electrospinning. These coated yarns were subsequently wound around elastic wires and woven into a vast expanse of electronic skin [Figure 5e]. The spiral structure of the GO‐doped PU nanofiber/Ni‐coated cotton yarn elastic composite yarn (GO‐PNF/NiCY elastic composite yarn) imparts stretchability to the sensor. This particular sensor can detect objects at a distance of ≈10 cm [Figure 5f].^[^ ^68^ ^]^

**Figure 6:**
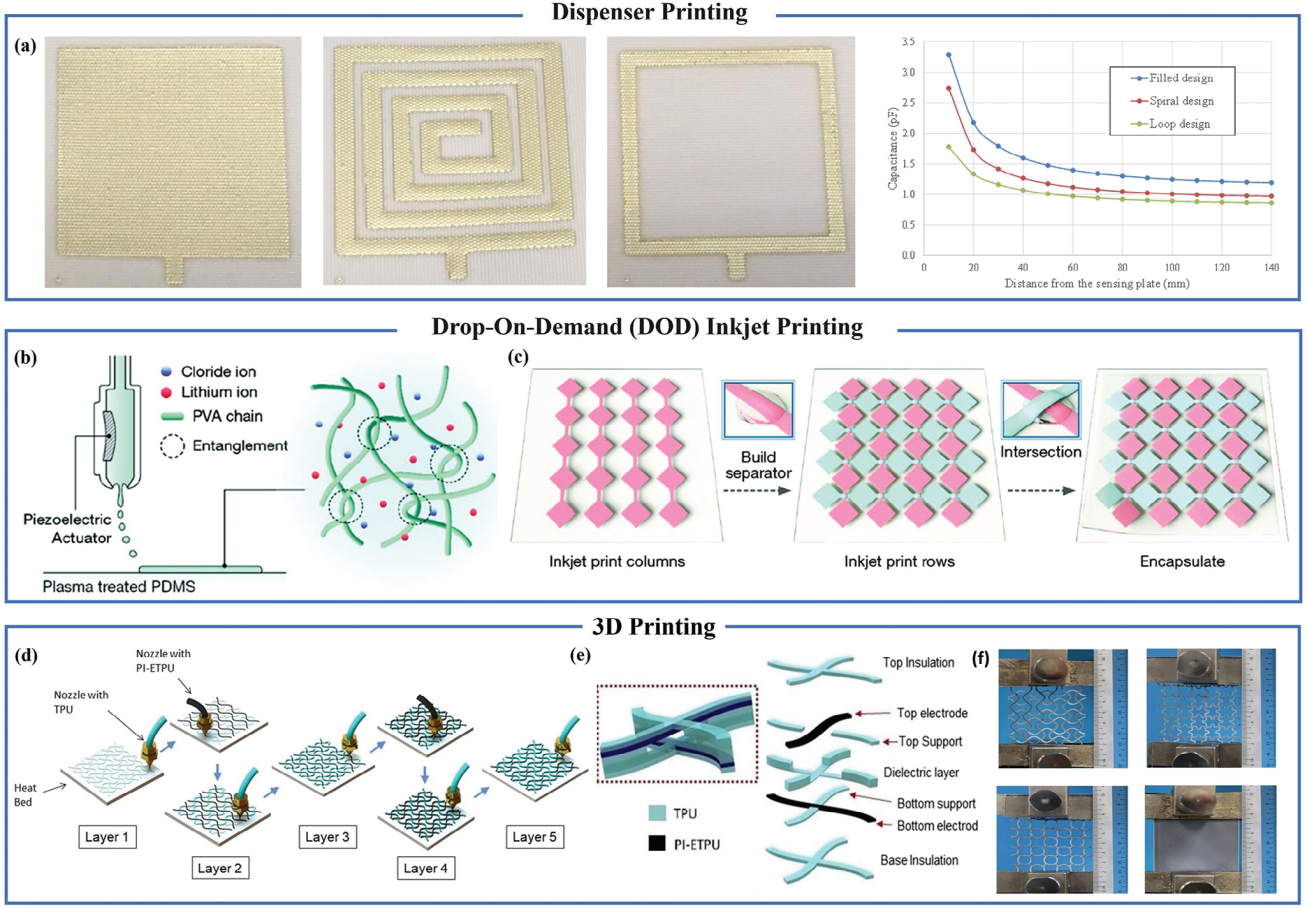
Large area flexible proximity sensor based on printing. a) Filled, spiral and loop proximity sensors and relative capacitance change with distance. Reproduced with permission.^[^ ^70^ ^]^ Copyright 2016, Elsevier. b) Schematic diagram of ink‐jet printing process and ion gel formation. c) Manufacturing process flow of stripe field capacitive touch sensing matrix with interlocking diamond electrode layout. Reproduced under terms of the CC‐BY license.^[^ ^71^ ^]^ Copyright 2020, The Authors, published by Wiley‐VCH. d) FDM 3D printing process diagram of sensor array manufacturing. e) Sensor structure diagram. f) Negative range, neutral range, positive range and uniform planar sample. Reproduced with permission.^[^ ^72^ ^]^ Copyright 2021, Wiley‐VCH.

Leveraging the benefits of waste reduction and operational simplicity, Li and his research team opted for inkjet printing to fabricate proximity sensors utilizing silver nanoparticle ink. The sensor structure comprises 100 grids.^[^ ^69^ ^]^ On the other hand, Yang Wei et al. utilized a dispenser printing method to create sensors on 100% polyester fabric. **Figure** **6**a illustrates sensors with three distinct structures, and simulations were conducted to analyze the variation of capacitance with distance. Experimental research revealed that, in the loop design of capacitive proximity sensors, when the ratio of track width (L1) to total electrode width (L) is 1/16, the usage of conductive ink was reduced by 76%, while still achieving 90% of the maximum detection distance.^[^ ^70^ ^]^ The choice of this ratio saves resources without affecting the performance of the sensor, achieving a win‐win effect. Additionally, it provides an idea for designing multimodal flexible sensors in the future, where the space occupied by proximity sensors can be limited to just the boundaries of the sensor. Dace Gao et al. used inkjet printing to print lithium chloride ink to form a coplanar interlocking diamond structure and fabricated sensors with transparent, elastic, and strain‐insensitive by 3D printing intermediate cross‐linked PDMS spacers [Figure 6b,c]. The sensor's detection distance is ≈100 mm.^[^ ^71^ ^]^ Meanwhile, Leon Yeon Wei Loh et al. utilized a commercial fused deposition modeling (FDM) multi‐material 3D printer to 3D print conductive carbon black thermoplastic polyurethane (PI‐ETPU) electrodes and insulating thermoplastic polyurethane (TPU) [Figure 6d]. During the printing process, the dual‐nozzle printer (BCN3D) intermittently changed the nozzles. The sensor's structure is depicted in Figure 6e. Figure 6f shows the potential for customizing Poisson's ratio by printing soft capacitive sensor arrays associated with three different metamaterial designs, which are in the auxetic, neutral, and positive ranges, respectively.^[^ ^72^ ^]^ Our bodies or humanoid robots possess 3D, irregularly shaped curves. Many existing processes for electronic skin are currently constrained by their planar nature. To address this issue, one solution is to create flat electronic skin using conventional or flat printing techniques and then apply it to a 3D surface. An alternative approach involves 3D printing technology, while the third method utilizes conventional large‐area coating techniques such as brush coating or spray coating to apply sensing materials to 3D surfaces.^[^ ^73^ ^]^

### 3.2 Multifunctionality

One of the remarkable features of human skin is its multifunctionality, encompassing a diverse array of sensory receptors that serve to detect pressure, strain, temperature, pain, vibration, and more.^[^ ^74^ ^]^

**Figure 7:**
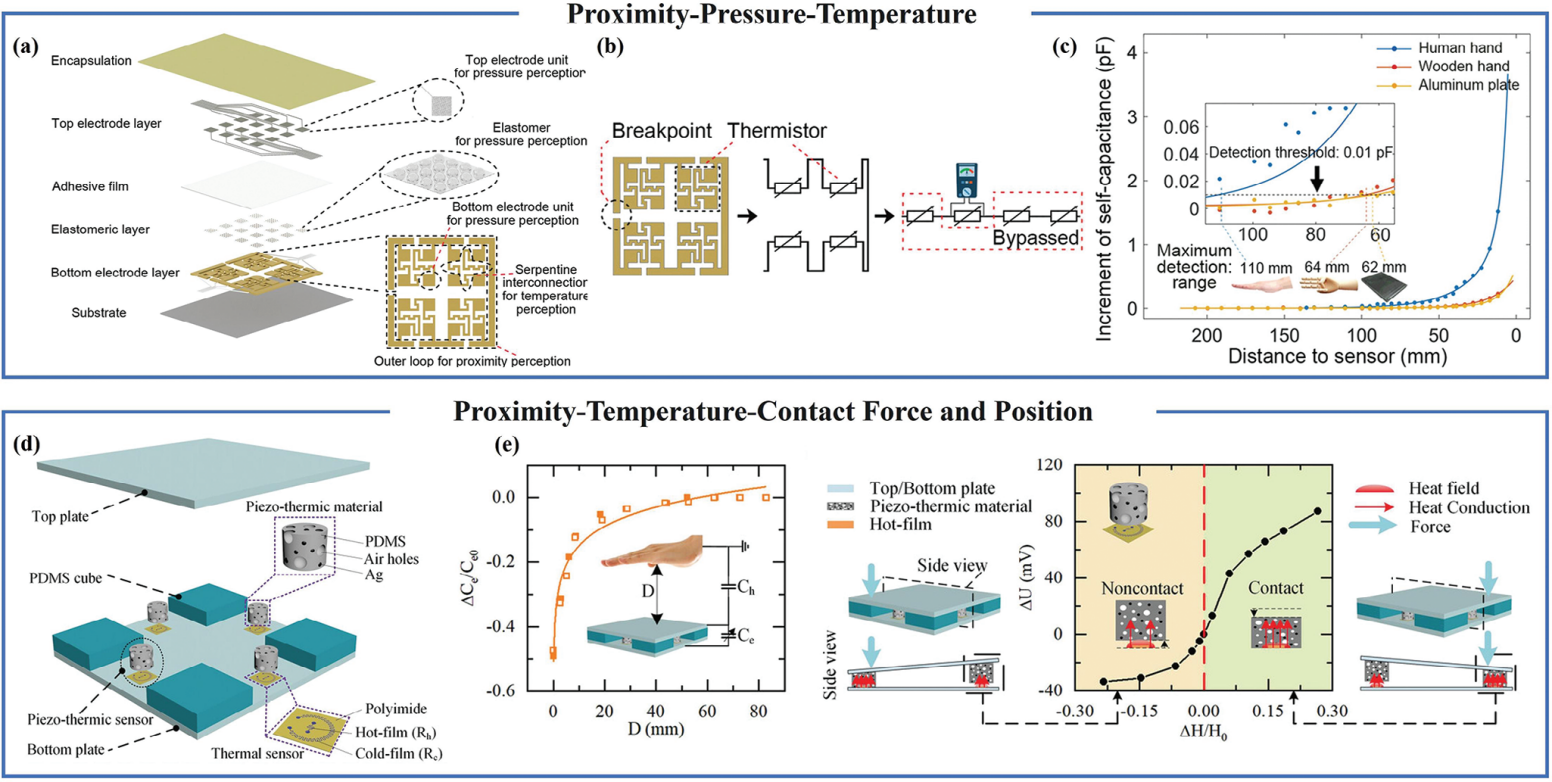
Multifunctional flexible proximity sensors with four or fewer functions. a) Exploded view of proximity, temperature and pressure sensors. b) Bottom electrode layer and its circuit schematic diagram under temperature detection mode. c) Changes in self‐capacitance when aluminum plates, wooden hands, and human hands approach. Reproduced with permission.^[^ ^77^ ^]^ Copyright 2021, Wiley‐VCH. d) Structural diagram of multifunctional electronic skin. e) Experimental response of piezoelectric thermal sensor to roof offset and tilt. Reproduced under terms of the CC‐BY license.^[^ ^79^ ^]^ Copyright 2022, The Authors, published by Wiley‐VCH.

Electronic skin, also known as e‐skin, is an electronic device or system designed to emulate the functionality of human skin. E‐skin should be considered as a versatile sensor capable of bridging the gap between humans, robots, and their surrounding environment. Recently, there has been a significant focus on the research and development of versatile flexible sensors with the capability to detect multiple external stimuli through various modes.^[^ ^75^ ^]^ These flexible, multifunctional sensors hold a pivotal role in the domain of electronic skin, facilitating seamless interaction between robots, humans, and their environment.^[^ ^76^ ^]^ Luo and his team harnessed the power of printing technology to integrate three essential functions‐proximity, temperature, and pressure‐into a single sensor, as illustrated in **Figure** **7**a. The proximity sensing capability of this sensor relies on self‐capacitive detection, with the range determined by the size of the surrounding boundaries. Therefore, silver ink is printed around the bottom sensor as a proximity sensor. This sensor consists of 16 square electrodes, forming a pressure sensor, and temperature sensors composed of thermistors interconnected in a serpentine pattern, as depicted in Figure 7b. The utilization of printing technology lays the groundwork for future large‐scale applications. This sensor can detect the proximity distance of a person's hand, ≈110 mm, as shown in Figure 7c.^[^ ^77^ ^]^ Moreover, the independent design of each sensor within this structure minimizes cross‐interference. Typically, pressure sensor electrodes or electrolytes are connected using materials with a high Young's modulus, leading to the propagation of deformation from one capacitive pressure sensor to another, causing interference.^[^ ^78^ ^]^ Li et al. developed a multimodal sensing electronic skin, as depicted in Figure 7d,e, which can detect proximity, contact force, contact force position, and temperature. They also proposed an intelligent robot control strategy to enable human‐machine interaction. The electronic skin employs parallel plate capacitors for proximity sensing, a pressure‐thermal sensor consisting of a mixture of PDMS, pores, and silver nanoparticles for contact force and position sensing, and a platinum/chromium (Pt/Cr) sensor for temperature detection. By utilizing a neural network, the sensor can determine contact force and contact position coordinates.^[^ ^79^ ^]^

**Figure 8:**
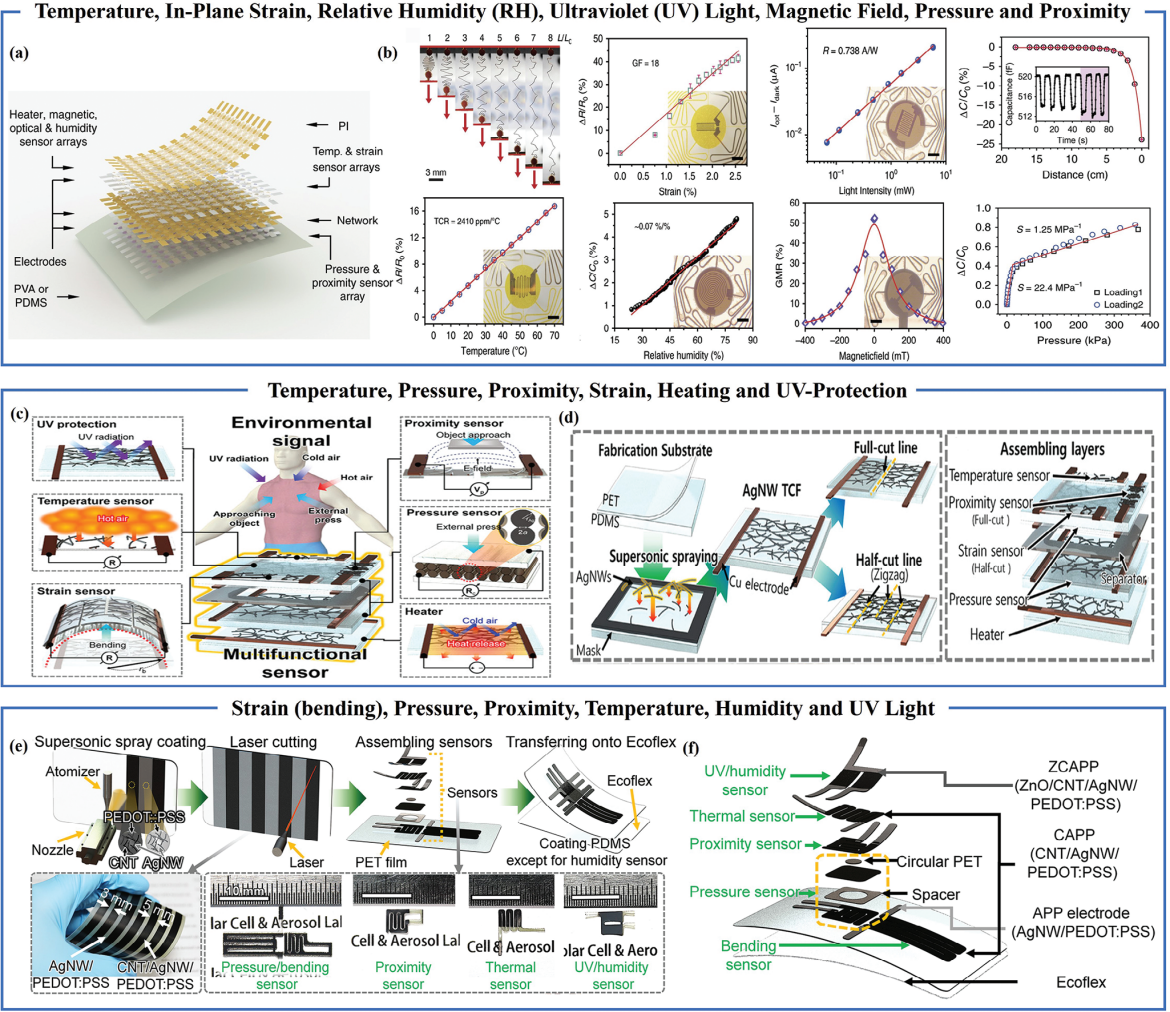
Multifunctional flexible proximity sensors with more than four functions. a) Layout schematic of SCMN‐an integrated sensor array with eight functionalities. b) Multifunctional sensing. Reproduced under terms of the CC‐BY license.^[^ ^85^ ^]^ Copyright 2018, The Authors, published by Springer Nature. c) Figure displaying a transparent flexible multifunctional sensor for detecting environmental stimuli. d) Multifunctional sensor assembly diagram. Reproduced with permission.^[^ ^86^ ^]^ Copyright 2019, American Chemical Society. e) Manufacturing process of multi‐functional soft sensors with pressure, bending, proximity, heat, light and humidity sensing functions. f) Overall structure of the sensor. Reproduced under terms of the CC‐BY license.^[^ ^87^ ^]^ Copyright 2022, The Authors, published by Springer Nature.

Typically, multifunctional sensors are designed as pressure‐proximity sensors^[^ ^80^, ^81^, ^82^ ^]^ or pressure‐proximity‐humidity sensors,^[^ ^83^, ^84^ ^]^ offering capabilities limited to two or three functions. However, there have been few reports on single sensors capable of detecting multiple stimuli. Hua and his research team introduced a highly stretchable and con‐formable matrix network (SCMN) composed of 100 sensory nodes interconnected by meandering wires, as depicted in **Figure** **8**a. This sensor mimics the functionalities of the human somatosensory system, encompassing capabilities to sense strain, pressure, temperature, and humidity. Remarkably, it incorporates functionalities beyond those of the human body, including light, magnetic field, and proximity sensing, as shown in Figure 8b.^[^ ^85^ ^]^ However, the fabrication process for this sensor is intricate and time‐consuming, resulting in elevated costs and limitations regarding high transparency and large‐scale applications. On the other hand, Hong Seok Jo and colleagues engineered a stretchable and transparent multifunctional sensor using supersonic‐sprayed silver nanowires (AgNW). This sensor is capable of monitoring pressure, proximity, strain, temperature, heating, and offering UV protection, as depicted in Figure 8c. The manufacturing process and the sensor's structure are delineated in Figure 8d, incorporating a silver nanowire transparent conducting film (AgNW TCF) to create proximity, pressure, and temperature sensors. During production, the AgNW TCF in the central segment is wholly severed and employed as a proximity sensor, while the AgNW TCF with a single notch on one side serves as a temperature sensor. Experimental findings revealed that as the number of supersonic spray scans increases, the sensor's transparency gradually diminishes, along with a reduction in sensitivity to pressure and proximity.^[^ ^86^ ^]^ Recently, Hong Seok Jo and colleagues introduced a sensor prepared using the supercritical spray method, endowed with the ability to simultaneously detect proximity, strain, pressure, temperature, humidity, and ultraviolet (UV) light, vividly portrayed in Figure 8e,f. The design incorporates a multi‐layer structure to prevent interference between different functions. Proximity sensing is achieved through fringe capacitance, offering non‐contact 3D scanning capabilities. Notably, the sensor demonstrates an ability to detect objects at a distance of ≈100 mm.^[^ ^87^ ^]^ By amalgamating the detection of six external stimuli within a single sensor, this advancement simplifies the layout of sensors in subsequent multi‐modal sensor systems.

### 3.3 Strain

**Figure 9:**
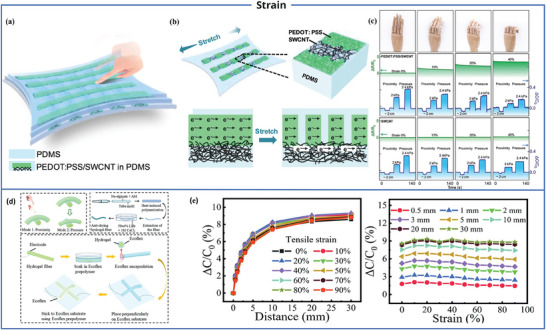
Strain performance of proximity sensor. a) Based on PEDOT: PSS/SWCNT proximity sensor structure. b) Electrical conductivity diagram before and after stretching. c) Pressure/proximity sensing under distinguishable strain. Reproduced with permission.^[^ ^89^ ^]^ Copyright 2020, American Chemical Society. d) Schematic diagram of a capacitive fiber optic bimodal sensor and its manufacturing process. e) Proximity response during tensile strain process. Reproduced with permission.^[^ ^90^ ^]^ Copyright 2022, The Royal Society of Chemistry.

One of the typical characteristics of human skin is its excellent adaptability to strain. Skin exhibits remarkable flexibility, allowing it to bend, stretch, or compress under external pressures or deformations. Although stretchable flexible proximity sensors have been developed,^[^ ^88^ ^]^ most of the reported responses of flexible proximity sensors are still measured under flat and strain‐free conditions, which, to some extent, limits their performance in real‐world applications. In order to better enhance the application potential of flexible proximity sensors, measurement experiments should not only be conducted under flat conditions, but also in bending and other states, and even achieve object positioning function in these states. This advancement will enhance the performance and applicability of flexible sensors in various scenarios and pave the way for their broader use in practical applications. One approach is to determine the stretching state when stretching alters the sensing signal and then determine the pressure or proximity corresponding to the output signal of the sensor. Zhao and his colleagues developed a hybrid electrode based on a transparent polymer (poly (3,4‐ethylenedioxythiophene): poly (styrene sulfonate) (PEDOT: PSS)/single‐walled carbon nanotubes (SWCNT)), embedded in a bottom PEDOT: PSS/SWCNT hybrid electrode array of PDMS packaging layer, and a sensor composed of a PDMS dielectric layer [**Figure** **9**a]. Under no strain, charge transport is dependent on PEDOT: PSS, whereas under significant strains, charge transport relies on SWCNT [Figure 9b]. As shown in Figure 9c, the resistance signal is influenced only by stretching and not by pressure or proximity. The sensor can initially ascertain the bending state through changes in resistance, and subsequently accurately determine the pressure and distance based on the capacitance's dependence on object pressure and proximity in a fixed bending state.^[^ ^89^ ^]^ Ding et al. synthesized hydrogel fibers using acrylamide (AAm) and sodium alginate as raw materials. These fibers were subsequently encapsulated in an Ecoflex layer, with two fibers vertically intersecting to create a sensor [Figure 9d]. This sensor displays outstanding attributes, with virtually no alteration in proximity sensing performance under strain conditions of 0–100% and minimal influence on proximity sensing output signal under stretching conditions of 0–90% [Figure 9e]. In the case of stretching, the proximity response of the sensor remains essentially unchanged, and this can be attributed to the essentially unchanged values of the sensor's initial capacitance, the capacitance of the object, and the distance between the object and the sensor. As the distance (d) and the contact area (s) of the capacitor both decrease to a similar extent during the stretching process, this may result in the sensor's initial capacitance remaining essentially unchanged. Additionally, the sensor features a broad detection range of 18 cm and is devoid of crosstalk between pressure and proximity sensing.^[^ ^90^ ^]^

### 3.4 Self‐Healing

**Figure 10:**
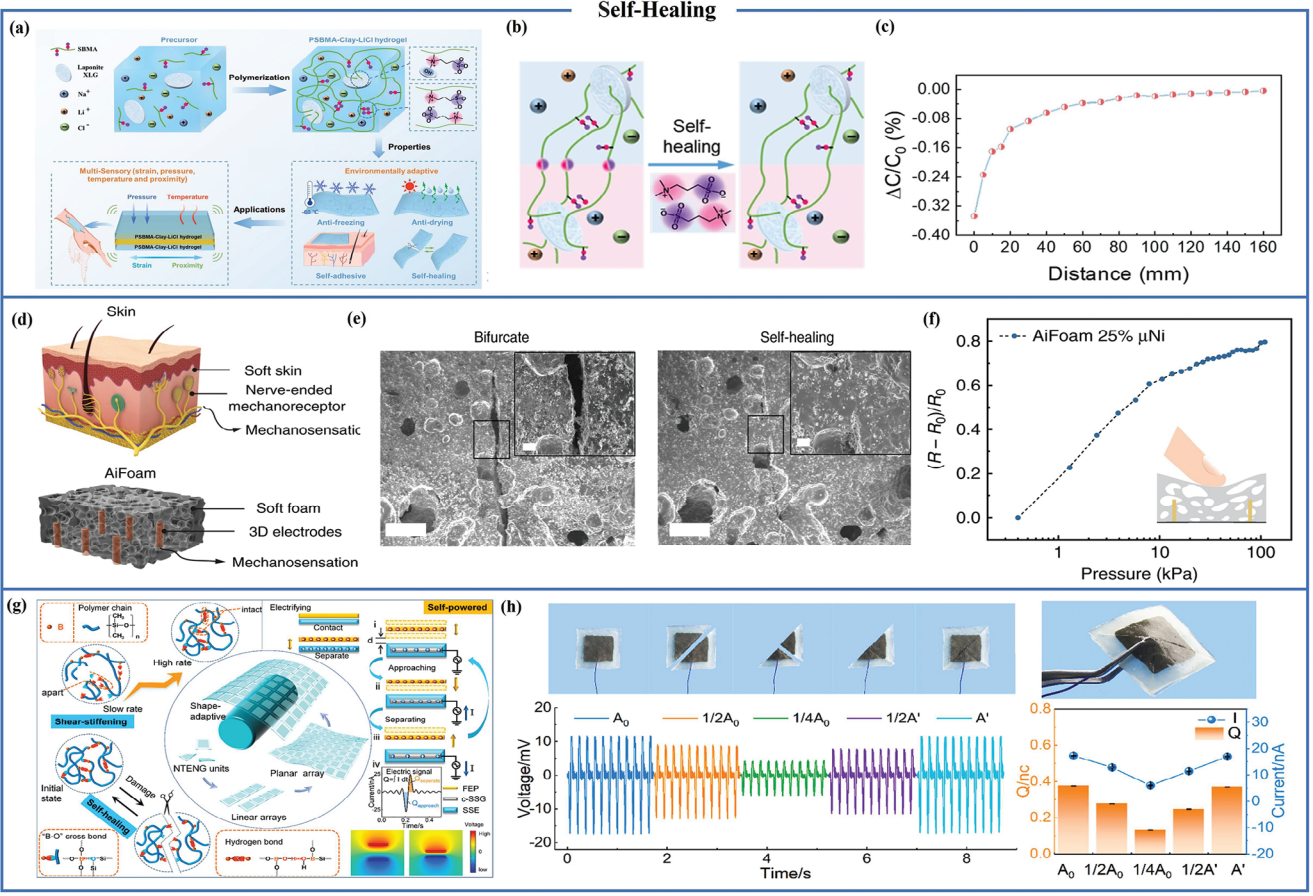
Self‐healing performance of proximity sensor. a) Schematic diagram of synthesis, properties and applications of PSCL hydrogel. b) Self‐healing mechanism of PSCL hydrogel. c) Relative capacitance changes with finger proximity distance. Reproduced with permission.^[^ ^94^ ^]^ Copyright 2022, Elsevier. d) Sensor structure diagram based on AiFoam. e) Self‐healing performance of AiFoam. f) Capacitance change response of distance between finger and sensor (with 25 µ Volume percentage of Ni). Reproduced under terms of the CC‐BY license.^[^ ^95^ ^]^ Copyright 2020, The Authors, published by Springer Nature. g) The assembly and self‐healing mechanism of NTENG. h) Digital photos of cutting and repairing units, as well as corresponding non‐contact electrical performance. Reproduced with permission.^[^ ^96^ ^]^ Copyright 2021, Elsevier.

One of the skin's most remarkable features is its self‐healing ability, which enables it to repair damage. Human skin possesses sufficient strength to self‐heal when subjected to injuries. In contrast, artificial electronic devices can deteriorate over time and may even fail due to factors such as fatigue, corrosion, or physical damage.^[^ ^91^ ^]^ Introducing self‐healing materials can provide sensors with self‐healing properties, thereby extending their lifespan and offering important advantages in the field of sensors.^[^ ^92^, ^93^ ^]^ Chen and colleagues used inorganic nanoclay and lithium chloride to synthesize a PSBMA‐Clay‐LiCl (PSCL) hydrogel through in situ polymerization of the polyelectrolyte. They then sandwiched this hydrogel between VHB sheets to create a sensor with both mechanical and electrical self‐healing capabilities, as illustrated in **Figure** **10**a. The self‐healing mechanism of this sensor is delineated in Figure 10b. When two freshly cut hydrogel pieces come into contact, an initial physical connection is established, followed by an accelerated healing process facilitated by ion interactions. This sensor is capable of detecting objects at a range of ≈160 mm, as depicted in Figure 10c.^[^ ^94^ ^]^ Guo et al. and their collaborators proposed a new structure. They embedded 3D electrodes into the new self‐healing foam material (AiFoam) [Figure 10d]. The robust dipole‐dipole interaction between surfactant molecules and the cross‐linked polymer network effectively traps the surfactant within the underlying elastomer, enabling the foam material to swiftly self‐heal and regain functionality after sustaining mechanical damage [Figure 10e]. By embedding a 3D electrode into a foam containing µNi particles, it can form a capacitive sensor and detect objects within ≈12 cm [Figure 10f].^[^ ^95^ ^]^ Yuan and colleagues introduced a self‐powered non‐contact triboelectric nanogenerator (NTENG) based on electrostatic induction and the triboelectric effect. This device incorporates graphene/shear stiffening gel (SSG) electrodes and a shear stiffening elastomer (SSE) shell. Notably, the sensor possesses the capability to self‐heal, dissipate, and absorb impact energy. It achieves self‐healing by leveraging cross‐links and dynamic hydrogen bonds within the polymer. These dynamic bonds can break during cutting but re‐solidify during the healing process, all without the need for external stimuli. When the NTENG is cut and subsequently healed, its output voltage, current, and charge remain consistent with the pre‐cut state, with the healing process taking just one minute, as depicted in Figure 10g,h. Additionally, this sensor can detect FEP films of ≈8 cm in size.^[^ ^96^ ^]^

## 4 Dual Type Proximity Sensor

**Figure 11:**
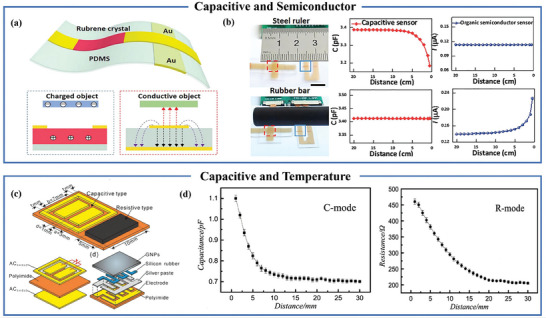
Dual type proximity sensor. a) Structural diagram of flexible conformal composite proximity sensor. b) Proximity response of dual types of sensors to steel rulers and charged rubber rods. Reproduced with permission.^[^ ^100^ ^]^ Copyright 2021, Elsevier. c) Structure of capacitive and resistive proximity sensors. d) Changes in capacitance (C‐mode) and resistance (R‐mode) of a 60 **°C** constant temperature plastic block at different distances from the sensor during proximity induction testing. Reproduced with permission.^[^ ^101^ ^]^ Copyright 2015, IOP Publishing Ltd.

Currently, many studies are focused on single types of proximity sensors, but they often fail to meet the requirements or achieve the desired results in certain situations. The integration of diverse proximity sensor types offers the potential to attain a spectrum of intricate functions and further enhance sensor performance. While existing systems combine flexible proximity sensors with various sensor types such as triboelectric and piezoresistive sensors,^[^ ^97^ ^]^ or triboelectric and piezoelectric sensors,^[^ ^98^ ^]^ they predominantly rely on a singular proximity sensing principle. However, by effectively harnessing the principles and characteristics of various flexible proximity sensors, there lies an opportunity for substantial improvement in proximity sensing performance. This integration and optimization harbor the potential to drive notable advancements in proximity sensor technology, thereby bolstering its capabilities across a multitude of applications. The inductive mode exhibits the capability to distinguish metal materials based on their distinct permeabilities, while the capacitive mode discriminates non‐metallic materials grounded in their dielectric constants. In daily life, insulating materials like hair, paper, and plastic sheets often accumulate charges imperceptibly, and flexible semiconductor proximity sensors excel at detecting these insulating materials. By integrating flexible capacitive sensors with semiconductor sensors, it is possible to address the limitations associated with the tested materials and simultaneously detect conductors and insulators.^[^ ^99^, ^100^ ^]^ Zhao et al. used andalusite crystal material as the sensing layer of organic semiconductor proximity sensors and gold electrode as the electrode of the sensor [**Figure** **11**a]. Experimental results show that the capacitive signal and the current signal have different detection capabilities and response characteristics. The capacitive signal has a detection range of 0.5 to 6 centimeters, while the current signal's detection range extends from 0.5 to 10 centimeters. These differences are due to the different detection principles. Figure 11b illustrates the response of the capacitive proximity sensor to a steel ruler, while the organic semiconductor sensor responds to a charged rubber rod.^[^ ^100^ ^]^ Furthermore, combining capacitive and resistive sensing units allows for the full utilization of the high sensitivity and rapid response characteristics of capacitive proximity sensors at close distances. This approach also lever‐ages the advantages of resistive temperature sensors in detecting objects over larger distances. Qiu et al. adopted the approach of embedding a capacitive sensor with coplanar electrodes and an interdigital temperature sensor with graphene nanosheets as the temperature sensing material into polyimide to create a dual‐type proximity sensor [Figure 11c]. This combination of capacitance and resistance engenders more efficient collaborative object proximity detection [Figure 11d].^[^ ^101^ ^]^ Additionally, sensors grounded in other principles can complement capacitive proximity sensors, for instance, the integration of capacitive sensors with inductive sensors enabling the detection of various materials.^[^ ^102^ ^]^

## 5 Application

In recent years, proximity sensors have seen widespread adoption across various domains of life. For instance, capacitive proximity sensors have been utilized in tasks such as liquid level detection,^[^ ^103^ ^]^ assessing the aging of composite insulators,^[^ ^104^ ^]^ material identification,^[^ ^105^ ^]^ and measuring automotive strain. The rapid advancements in artificial intelligence technology have driven the development of functional sensors, particularly within the realm of smart sensing technology. This technology empowers both humans and robots to swiftly and accurately gather information from their external environments, reshaping people's lives and providing convenience across various domains. Research into electronic skin not only contributes to enhancing the intelligence of robots but also ensures safety in human‐robot collaborations. The integration of flexible proximity sensors into smart environments has introduced a captivating dimension to life. For instance, in everyday life and entertainment, these sensors find application in gesture detection and interaction.

### 5.1 Human–Robot Collaboration

**Figure 12:**
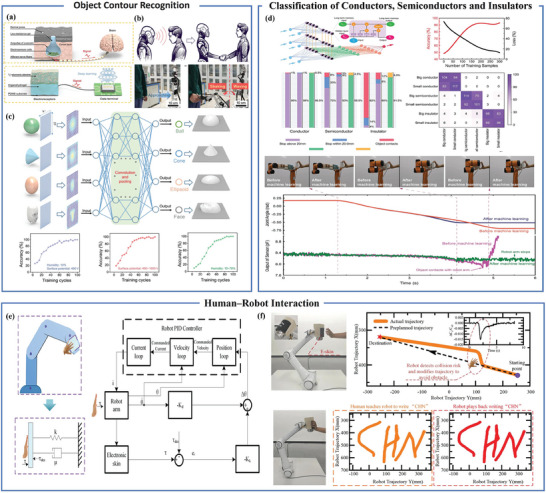
Flexible proximity sensors applied in human‐robot collaboration. a) Shark‐inspired electroreceptor and artificial electroreceptor. b) Demonstration of manipulating robot arm when an adult is approaching. c) Target recognition of cones, cylinders, ellipses, and other objects based on electric receptor systems. Reproduced under terms of the CC‐BY license.^[^ ^108^ ^]^ Copyright 2022, The Authors, published by AAAS. d) Improving the approaching process performance of the multi‐functional electronic skin system through machine learning. Reproduced under terms of the CC‐BY license.^[^ ^110^ ^]^ Copyright 2023, The Authors, published by Wiley‐VCH. e) Schematic diagram of robot feedback control system for collision detection and safety control. f) Applying electronic skin on robot arms to achieve human‐machine interaction and teaching. Reproduced under terms of the CC‐BY license.^[^ ^79^ ^]^ Copyright 2022, The Authors, published by Wiley‐VCH.

With the relentless progress of science, technology, and the rapid advancements in artificial intelligence, humanity has ushered in the era of intelligence. In recent times, the swift advancement of mobile internet and intelligent gadgets has substantially stimulated the investigation of smart sensing technologies in areas like human‐robot interaction, machine learning, and portable devices. Cutting‐edge flexible sensor technology for robots is achieving remarkable breakthroughs, contributing significantly to the emergence of the intelligent era. While the precise origin of the term “Fifth Industrial Revolution” (5IR) remains somewhat un‐clear, it emphasizes human‐robot collaboration over competition.^[^ ^106^ ^]^ Security is a paramount concern in the future of human‐robot collaboration. Furthermore, vision‐based sensors may not function optimally in certain scenarios. To address these challenges, the application of proximity sensors assumes particular significance. Enhancing the safety of human‐computer interactions in industrial environments can be achieved through distance detection, surface contouring, and motion tracking.^[^ ^107^ ^]^ In the depths of the ocean, sharks employ electrical reception strategies for remote sensing, and machine learning holds the promise of extending functionality with minimal sensor deployment. As depicted in **Figure** **12**a, based on these principles, Guo et al. developed an artificial electroreceptor that incorporates electret‐embedded inorganic nanoparticles SiO_2_, ionic hydrogel, and PDMS elastomer. Figure 12b illustrates this sensor attached to a robot's surface, enabling interaction through the electrical charge naturally carried by humans. Utilizing the data‐to‐color approach, the potential distribution is translated into a 2D image, facilitating enhanced feature extraction by the convolutional neural network. Ultimately, the VGG‐16 architecture, known as the Visual Geometry Group, provided the foundational framework for the convolutional neural network, achieving recognition of spheres, cones, ellipses, and human faces with an impressive accuracy rate of 97% [Figure 12c].^[^ ^108^ ^]^ Gege Ma and colleagues have pioneered a 4D sensing technique in conjunction with a spatio‐temporal fully variational algorithm. This innovation employs 4D mapping to enable object tracking, subsequently facilitating the development of predictive models to control robot movement based on obstacle trajectories. This is particularly valuable for robots operating in unpredictable environments and seeking to avoid collisions.^[^ ^109^ ^]^ Ge et al. integrated their multifunctional electronic skin into a commercial robotic arm and found that insulating objects did not trigger an immediate response when approaching the robotic arm. This is because insulators cannot modify capacitance as effectively as conductors, causing the proximity sensor's output to fall short of the active safety control threshold. To address this issue, a Long Short Term Memory (LSTM) network for object classification was used. The network achieved exceptional accuracy rates of 99.5%, 95.5%, and 91.5% [Figure 12d]. Through this network, the threshold was dynamically optimized in real‐time, thereby enhancing the effectiveness and precision of active safety control.^[^ ^110^ ^]^ Li et al. installed their designed electronic skin on the six degrees of freedom manipulator to achieve collision detection of robots. When the detected signal exceeds the predefined safety threshold, the robot will trigger its safety control to reduce damage. The author assumes that the robotic arm follows the spring damping dynamic mechanism and proposes the following interactive control laws to achieve human‐robot interaction of the robotic arm:

(1) $$\Delta \theta \;=-K_{k}e_{\tau }-K_{d}\dot{\theta }$$

where Δ ***θ*** is the angle increment of the robot, ***k***_***k***_ and ***k***_***d***_ are the coefficients associated with the stiffness and damping of the kinetic system, respectively, ***e***_***τ***_ =  ***τ*** − ***τ***_***des***_ is torque deviation, ***τ*** is the interaction torque on the robot detected by the e‐skin, ***τ***_***des***_ is a desired acting torque, and $\dot{\theta }$ is the robot angular velocity. When the human hand is close to the robot arm, the robot arm will immediately stop moving and enter the interaction mode. When the human hand touch is detected, the robot immediately enters the human‐robot interaction mode, and the control flowchart is shown in Figure 12e. By mounting it on the robotic arm, it can avoid obstacles and teach the robot to handwrite words and perform other operations [Figure 12f].^[^ ^79^ ^]^ Seung Jae Moon et al. employed dual‐type proximity sensors developed by Aidin Robotics to handle obstacles through admittance control and distance measurement techniques.^[^ ^111^ ^]^

In the enterprise setting, MRK System utilizes capacitive skin to implement collision avoidance functionality. Additionally, Aidin Robotics has developed dual‐type proximity sensors for compliant control in robotic arms. Fogale Robotics and Yuejiang Technology have successfully commercialized proximity‐sensitive safety skins, enabling obstacle avoidance in robotic arms. Proximity sensors can also be employed for object localization during the robotic grasping process.^[^ ^112^ ^]^

As future robotic work environments grow increasingly complex, there is a heightened demand for enhanced robot flexibility, safety, and other aspects. Single‐modal sensing technology has become inadequate to meet these contemporary requirements. Single‐modal perception can offer only partial insights into objects, rendering inferences about object images susceptible to misinterpretation.^[^ ^113^ ^]^ Consequently, it is imperative to introduce multimodal information in the future to comprehensively capture objectattributes and fulfill the operational demands of future robots in intricate environments.

### 5.2 Human Machine Interface

**Figure 13:**
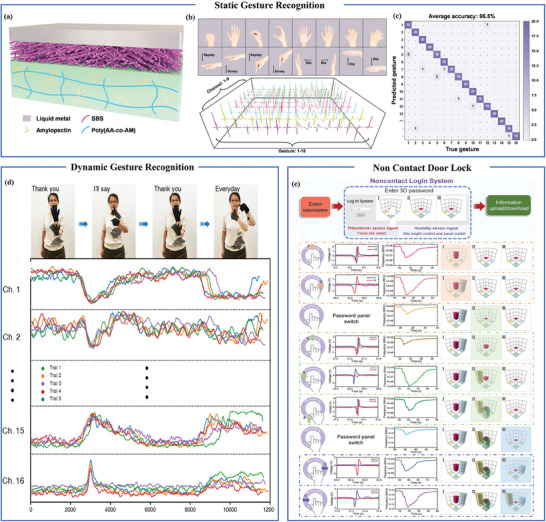
Flexible proximity sensors applied in human‐machine interface. a) Structural design of TTS (touchless tracking system). b) Photographs of 16 different non‐contact gestures and electrical signals from the TTS. c) Confusion matrix for verifying the structure. Reproduced with permission.^[^ ^127^ ^]^ Copyright 2022, Wiley‐VCH. d) Display images of “Thank you”, “I'll say”, and “Everyday” dynamic gestures in sequence. Reproduced with permission.^[^ ^137^ ^]^ Copyright 2021, American Chemical Society. e) Login to the system's non‐contact 3D password interface. Reproduced under terms of the CC‐BY license.^[^ ^138^ ^]^ Copyright 2022, The Authors, published by Wiley‐VCH.

The human‐machine interface (HMI) plays a pivotal role in human‐computer interaction.^[^ ^114^ ^]^ Amid the pandemic, touchless HMI has garnered heightened interest due to its flexibility and hygiene advantages. It demonstrates substantial potential in various domains, encompassing gesture recognition^[^ ^115^, ^116^, ^117^, ^118^ ^]^ and touchless door locks.^[^ ^119^ ^]^ Vision‐based gesture recognition is an effective method for non‐contact HMI. Its system is generally based on color cameras or depth sensors to collect gesture data.^[^ ^120^ ^]^ This information is then processed and analyzed, such as image segmentation, extraction and classification of gesture actions, etc.^[^ ^121^ ^]^ However, vision‐based gesture recognition has problems such as being affected by lighting (such as changing lighting conditions),^[^ ^122^, ^123^, ^124^, ^125^ ^]^ time‐consuming processing of large amounts of image data, and occlusion of complex gestures.^[^ ^123^, ^124^, ^125^, ^126^ ^]^ Proximity sensors used for gesture recognition have the advantages of not being affected by light and having a small amount of data, so using proximity sensors for gesture recognition has certain advantages. Zhou et al. combined triboelectric proximity sensors with deep learning to create an intelligent touchless gesture recognition system. The sensor comprises liquid metal, polymer fibers (SBS), and branched starch‐based hydrogel [**Figure** **13**a]. It can easily and securely adhere to various common substrates. They integrated the sensor with a deep learning‐based multilayer perceptron (MLP) neural network, achieving the recognition of 16 different hand gestures with an impressive average accuracy rate of 96.5% [Figure 13b,c].^[^ ^127^ ^]^ Wearable systems find extensive use across various applications, notably in health monitoring, virtual reality,^[^ ^128^ ^]^ and augmented reality.^[^ ^129^, ^130^ ^]^ Wearable gesture detection has been extensively researched, primarily based on resistive sensors,^[^ ^131^, ^132^ ^]^ electromyography (EMG),^[^ ^133^, ^134^, ^135^ ^]^ and optical devices.^[^ ^136^ ^]^ However, the majority of these studies focus on single‐modal systems. Pan et al. developed a flexible wearable system with a hybrid design to capture both spatial and temporal data from finger movements and hand positions. As shown in Figure 13d, peaks represent sudden tactile movements, while more gradual changes indicate approaching movements. The constructed bimodal sensor platform achieves high‐precision recognition of static and dynamic gestures (such as “Thank you”, “I'll say”, and “Everyday”) through machine learning and LSTM neural networks, respectively.^[^ ^137^ ^]^

In everyday life, door locks or entering passwords in bank are often accomplished using touch keypads. However, this method poses the risk of leaving fingerprints or viruses on the keypad, potentially resulting in the exposure of personal information and hygiene concerns. Touchless input technology can address this issue. Le et al. accomplished touchless password input by combining a piezoelectric resonant humidity sensor with a triboelectric proximity sensor. The humidity sensor consists of a two‐port aluminum nitride (AlN) body resonator and a uniformly thick graphene oxide film. The two‐port piezoelectric body wave resonator is fabricated on a layered structure of AlN piezoelectric layer and Si structure layer, with the Si layer highly doped and serving as the bottom electrode. The triboelectric proximity sensor consists of two parts, including a point base and an Ecoflex ring on the fingertip. As shown in Figure 13e, the input display interface for non‐contact passwords is composed of three interfaces that do not interfere with each other. There are four control points included in each input interface. The triboelectric‐based sensor is responsible for activating the panels and determining the position of the password. The humidity sensor controls the switching of the password panels and increases the number of panels. This approach enables touchless input of 3D passwords. Furthermore, flexible proximity sensors can also enable non‐contact control of games, providing players with a more immersive gaming experience.^[^ ^138^ ^]^

### 5.3 Remote Monitoring

**Figure 14:**
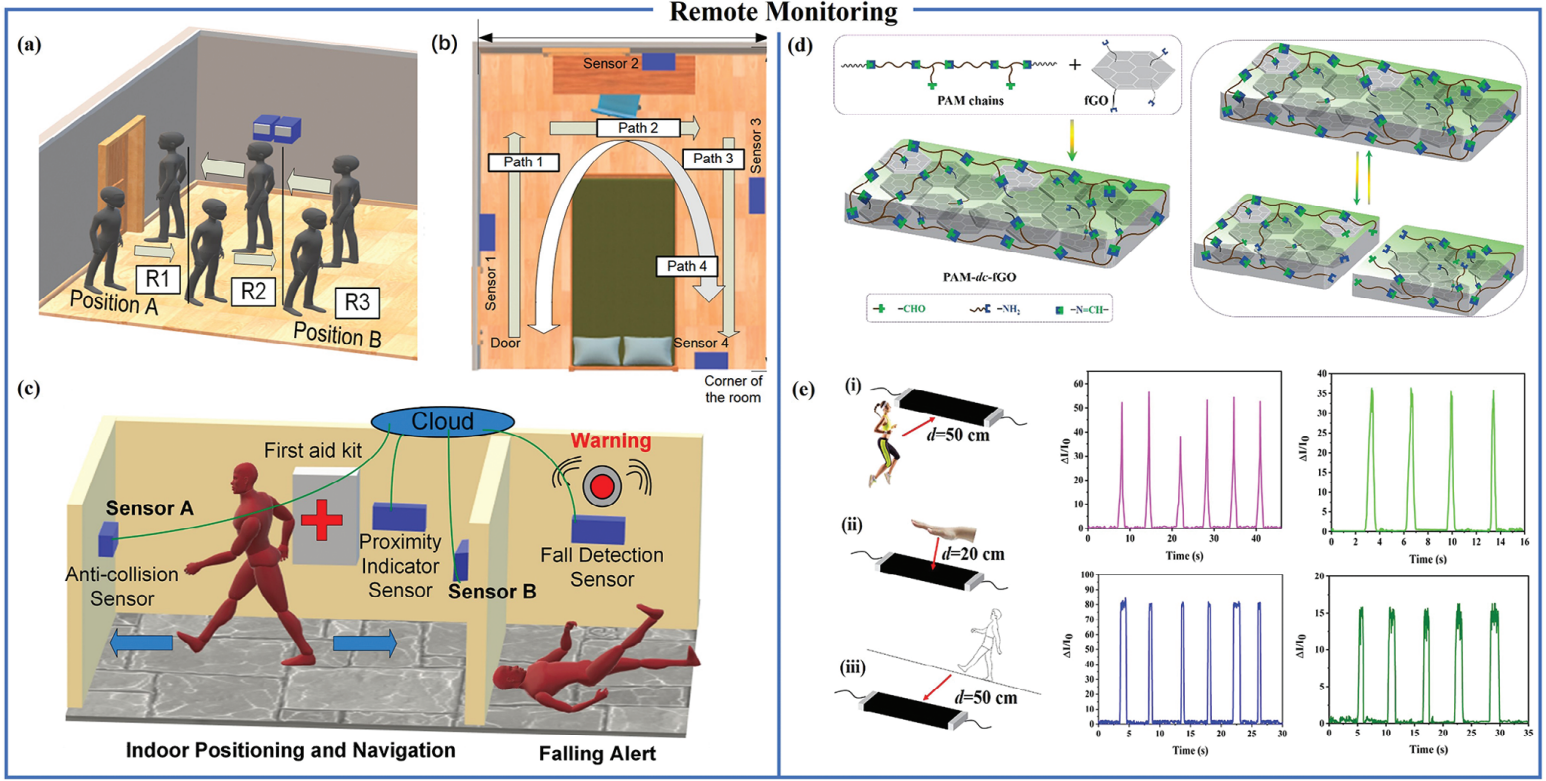
Flexible proximity sensors applied in remote monitoring. a) Walking and running direction monitoring. b) Indoor positioning system monitoring. c) Blind navigation assistance, indoor positioning and fall sensor detection. Reproduced with permission.^[^ ^140^ ^]^ Copyright 2021, Elsevier. d) Preparation and self‐healing properties of PAM‐dc‐fGO thin films. e) Remote monitoring of human motion through film‐based proximity sensor. Reproduced with permission.^[^ ^141^ ^]^ Copyright 2020, American Chemical Society.

Monitoring human movement and its interaction with the surrounding environment is of particular importance for medical applications.^[^ ^139^ ^]^ Proximity sensors are extensively employed in safety prevention and healthcare systems, facilitating real‐time and precise monitoring of movement without direct contact with the human body. This provides favorable conditions for remote monitoring. In medical facilities or elderly care environments, individuals’ memory impairment or resistance to wearable devices may affect the effectiveness of monitoring based on such devices. Therefore, an ideal medical monitoring device should not only fulfill its monitoring function but also respect the privacy of the individuals being monitored. Anaya et al. have engineered a triboelectric‐based sensor by connecting a negatively charged precharged PDMS film to aluminum (Al) foil. This sensor enables near‐field remote behavior monitoring of the human body within a range of 1.5 meters. It can estimate the relative positions of two individuals and distinguish between various human activities, such as jumping and walking. As shown in **Figure** **14**a, the walking direction of a person can be identified using two sensors combined with a new signal power discrimination algorithm. In Figure 14b, four sensors are placed at fixed positions on the wall, allowing the walking path of individuals in the house to be obtained based on the time sequence of each sensor response. In Figure 14c, sensors are placed at specific locations, such as walls. When a blind person approaches, the voltage increases, and the positive and negative peak values reach the set threshold. The data is transmitted to the wristband alarm through Bluetooth, triggering the alarm to achieve a collision prevention function. The sensor can also be placed in key dangerous places, such as shower doors. When someone falls in front of the shower door, the sensor signal will suddenly have a negative voltage peak, followed by two positive voltage peaks. This will be judged as a fall by the code, and then the alarm will sound an alarm, thereby achieving fall detection.^[^ ^140^ ^]^ Furthermore, Zhang et al. developed a conductive film (PAM‐dc‐fGO) by cross‐linking intrinsic self‐healing polyazome‐thine (PAM) with ethylenediamine‐functionalized graphene oxide (fGO) through dynamic covalent bonds (imine bonds, −CH = N−) [Figure 14d]. After 24 h, the severed conductive film can self‐heal, and its stress‐strain curve matches the initial state. This conductive film can be integrated with the human body and air to create an organic field‐effect transistor (OFET), contributing to remote monitoring of the human body. While the human body is in motion, the conductive film translates a variety of movements into unique electrical signals, encompassing actions such as stomping, jumping, hand gestures, and walking back and forth in close proximity to the device. This capability enables the sensor to achieve remote monitoring of the human body [Figure 14e]. The sensor's detection range surpasses 50 cm, rendering it especially valuable in the domain of long‐distance monitoring.^[^ ^141^ ^]^ Moreover, flexible proximity sensors also have applications in remote positioning and other fields.^[^ ^37^ ^]^

## 6 Conclusion

In this review, we explore the most recent research findings on flexible proximity sensors. We place a strong emphasis on understanding the operational principles of capacitive proximity sensors and triboelectric proximity sensors. Additionally, the development of single type and dual type proximity sensors is summarized. The research progress of single‐type proximity sensors is described concerning large area, multifunctionality, strain, and self‐healing. Finally, the significance of flexible proximity sensors in applications such as human‐robot collaboration, human‐machine interface, and remote monitoring was summarized. With the development of flexible electronic technology, these sensors can achieve more intelligent and adaptive human‐machine interaction, providing key support for the future development of human‐machine collaboration.

Capacitive proximity sensors are deployable in non‐conductive shielded environments and offer a straightforward and cost‐effective sensor solution. However, factors such as the humidity level in the surrounding environment and the presence of internal objects can easily affect their detection accuracy. To mitigate this issue, one approach involves integrating capacitive proximity sensors with other detection methods, such as ultrasonic or inductive sensors. Alternatively, data processing techniques can be utilized to decrease data variability and minimize noise interference. Furthermore, specific sensor chips, such as the FDC2214 series chips, can aid in mitigating environmental interference.

On the other hand, triboelectric proximity sensors operate without requiring external power sources and demonstrate sensitivity to dynamic changes. However, their signal detection is instantaneous, which may have limitations in specific applications. Flexible proximity sensors hold great promise in the field of human‐robot collaboration. As ro‐bots increasingly take on diverse and complex tasks, the demand for high‐performance and adaptable flexible proximity sensors will continue to rise. However, along with opportunities come challenges.

1. *Multifunctionality*: Developing sensors with multiple integrated capabilities within a single device poses significant challenges. As mentioned previously, addressing inter‐ference between sensors and external stimuli and optimizing the distribution of sensor structures are critical factors.

2. *Self‐healing*: Research on the self‐healing properties of flexible proximity sensors remains relatively limited. Although self‐healing capabilities have been investigated in hydrogels or ion gels within electronic skins, a universally applicable implementation method is still lacking. Additionally, the practical implementation of self‐healing abili‐ties in real devices is still constrained.

3. *Response Time*: In applications such as robot safety, the response time of flexible proximity sensors plays a critical role. However, many research papers do not explicitly mention the response time related to proximity effects.

In addition to these challenges, addressing issues related to detection range, measurement accuracy, durability, and stability is essential to ensure the reliability and long‐term use of flexible proximity sensors. Furthermore, the effective integration of these sensors with robot systems for real‐time data transmission and processing, along with the extraction of valuable information from abundant sensor data, are areas of focus deserving careful consideration.

## Conflict of Interest

The authors declare no conflict of interest.
